# *MTHFR* 677C>T (rsRS1801133) variant is associated with hyperhomocysteinemia but not with clinical severity in patients with peripheral arterial disease

**DOI:** 10.1590/1677-5449.202200612

**Published:** 2023-11-20

**Authors:** Guilherme da Silva Silvestre, Iriana Moratto Carrara, Tamires Flauzino, Marcell Alysson Batisti Lozovoy, Rubens Cecchini, Edna Maria Vissoci Reiche, Andréa Name Colado Simão

**Affiliations:** 1 Universidade Estadual de Londrina – UEL, Londrina, PR, Brasil.; 2 Centro Universitário Filadélfia – UNIFIL, Londrina, PR, Brasil.; 3 Laboratório Central Diagnósticos de Londrina, Londrina, PR, Brasil.; 4 Universidade de São Paulo – USP, Ribeirão Preto, SP, Brasil.

**Keywords:** peripheral arterial disease, methylenetetrahydrofolate reductase, homocysteine, genetic variant, Fontaine classification, doença arterial periférica, metilenotetrahidrofolato redutase, homocisteína, variante genética, classificação de Fontaine

## Abstract

**Background:**

The *MTHFR* 677C>T variant’s involvement with hyperhomocysteinemia and peripheral arterial disease (PAD) is still unclear.

**Objectives:**

To evaluate associations between the *MTHFR* 677C>T (rs1801133) variant and susceptibility to and severity of PAD and homocysteine (Hcy) levels.

**Methods:**

The study enrolled 157 PAD patients and 113 unrelated controls. PAD severity and anatomoradiological categories were assessed using the Fontaine classification and the Inter-Society Consensus for the Management of Peripheral Arterial Disease (TASC), respectively. The variant was genotyped using real-time polymerase chain reaction and Hcy levels were determined using chemiluminescence microparticle assay.

**Results:**

The sample of PAD patients comprised 60 (38.2%) females and 97 (61.8%) males. Patients were older and had higher Hcy than controls (median age of 69 vs. 45 years, p<0.001; and 13.66 µmol/L vs. 9.91 µmol/L, p=0.020, respectively). Hcy levels and the *MTHFR* 677C>T variant did not differ according to Fontaine or TASC categories. However, Hcy was higher in patients with the CT+TT genotypes than in those with the CC genotype (14.60 µmol/L vs. 12.94 µmol/L, p=0.008). Moreover, patients with the TT genotype had higher Hcy than those with the CC+CT genotypes (16.40 µmol/L vs. 13.22 µmol/L, p=0.019), independently of the major confounding variables.

**Conclusions:**

The T allele of *MTHFR* 677C>T variant was associated with higher Hcy levels in PAD patients, but not in controls, suggesting a possible interaction between the *MTHFR* 677C>T variant and other genetic, epigenetic, or environmental factors associated with PAD, affecting modulation of Hcy metabolism.

## Resumo

**Contexto:**

O envolvimento da variante *MTHFR* 677C>T na hiperhomocisteinemia e na doença arterial periférica (DAP) ainda não está claro.

**Objetivos:**

Avaliar a associação da variante *MTHFR* 677C>T (rs1801133) com suscetibilidade e gravidade da DAP e valores séricos de homocisteína (Hcy).

**Métodos:**

Este estudo caso-controle envolveu 157 pacientes com DAP e 113 controles não relacionados. A gravidade e as categorias anatomorradiológicas da DAP foram avaliadas pela classificação de Fontaine e pelo *Inter-Society Consensus for the Management of Peripheral Arterial Disease*, respectivamente. A genotipagem foi realizada por meio de reação em cadeia da polimerase em tempo real, e os valores de Hcy foram determinados por ensaio de micropartículas de quimioluminescência.

**Resultados:**

Entre os pacientes com DAP, 97 (61,8%) eram homens e 60 (38,2%) eram mulheres, com mediana de idade de 69 anos. Os pacientes com DAP eram mais velhos e apresentaram valores mais elevados de Hcy do que os controles (mediana de 69 vs. 45 anos de idade, p < 0,001; 13,66 µmol/L vs. 9,91 µmol/L, p = 0,020, respectivamente). Os valores de Hcy foram mais elevados em pacientes com os genótipos CT+TT do que aqueles com o genótipo CC (14,60 µmol/L vs. 12,94 µmol/L, p = 0,008). Além disso, os pacientes com o genótipo TT apresentaram valores mais elevados de Hcy do que aqueles com os genótipos CC+CT (16,40 µmol/L vs. 13,22 µmol/L, p = 0,019, respectivamente), independentemente das principais variáveis confundidoras.

**Conclusões:**

O alelo T da variante *MTHFR* 677C>T foi associado a valores mais elevados de Hcy nos pacientes com DAP, mas não em controles, sugerindo uma possível interação entre a variante genética *MTHFR* 677C>T e outros fatores genéticos, epigenéticos ou ambientais associados com a DAP na modulação do metabolismo da Hcy.

## INTRODUCTION

Peripheral arterial disease (PAD) is a global public health concern; severe arterial stenosis and insufficient blood supply to the distal limb cause intermittent claudication and resting pain, reduce patients’ quality of life in terms of mobility and degree of independence, and can lead to disability and even death.^1^ In most cases it is characterized by atherosclerotic lesions involving non-cardiac and non-cerebral arteries and disturbances in the axial vessels and at the microcirculatory level, which may lead to critical ischemia of lower limbs. It is significantly associated with an increased risk for incident cardiovascular diseases, cerebrovascular diseases, and mortality.^2-5^

The underlying pathological mechanism of PAD and its potential pathways and core genes have received considerable research attention in recent years, aimed at identifying potential therapeutic targets^6^ and genetic variants through genome-wide association studies (GWAS) and epigenetic profiling.^7,8^ Better understanding of the genetics of PAD may allow earlier identification of those at risk of PAD and could open the door to precision medicine-based approaches to treatment.

Although familial aggregation and heritability estimates suggest a significant genetic contribution, little is known about the genetic susceptibility to PAD in non-European populations.^9-10^ GWAS of the ankle-brachial index (ABI) and PAD (defined as ABI < 0.90) showed that the variants associated with ABI differ by Hispanic/Latino ethnic groups.^11^

Homocysteine (Hcy) is normally present in the plasma in low concentration, typically in the range of 5 to 15 μmol/L (μM), and this variability may be related to different methodologies, differences in sampling processing, sex, and gender.^12^ Values higher than 15 μmol/L are identified as hyperhomocysteinemia (HHcy), a harmful condition and an independent risk factor for cardiovascular disease.^12,13^ The prevalence rates of HHcy are 5.0-7.0% in the general population^13^ and 28-30% in patients with PAD.^14^ A previous study has defined mild-moderate HHcy as Hcy concentrations in the range 10–100 µmol/L and severe HHcy as >100 µmol/L.^15^ HHcy is involved in endothelial dysfunction, an important step in atherosclerosis development,^13-16^ and a relation has been demonstrated between HHcy and PAD.^13,14^ The effects of mild-moderate HHcy were primarily vascular, including myocyte proliferation, vessel wall fibrosis, impaired nitric oxide signaling, superoxide generation, and pro-coagulant activity.^15,16^

Plasma Hcy concentration is influenced by a complex interaction of environmental and genetic factors.^17^ The *MTHFR* gene codes 5,10-Methylenetetrahydrofolate Reductase (MTHFR), a critical enzyme in remethylation of Hcy to methionine, and the 677C>T (rs1801133) variant of this gene is the most common genetic cause of HHcy. This variant consists of a C (cytosine) to T (thymine) transition at nucleotide 677 in exon 4.^18^ HHcy and the *MTHFR* 677 C>T genetic variant emerged as an independent risk factor for subclinical atherosclerosis, implying genetic influences may be potential contributors to the increased burden of atherosclerotic disease that characterizes PAD. The association between PAD and *MTHFR* variants has been studied in literature with controversial results; the Linz Peripheral Arterial Disease (LIPAD) study did not correlate *MTHFR* 677C>T with PAD,^18^ while a meta-analysis of nine appropriate studies showed that homozygosity for the T allele was associated with an increased risk of PAD.^19^

Although some studies have assessed *MTHFR* 677C>T variant’s involvement with HHcy and the development of PAD, data remain scarce and contradictory.^18-20^ Moreover, there are no studies that have evaluated involvement of the *MTHFR* 667C>T genetic variant with the severity of PAD in the Brazilian population. Since the results of studies carried out with other populations are quite diverse, it is necessary to achieve a better understanding of the genetic characteristics and behavior of PAD in our population. Considering the hypothesis that *MTHFR* 677C>T could be involved in the etiology and prognosis of PAD, the aim of the present study was to evaluate associations between the *MTHF*R 677C>T variant and susceptibility to and severity of PAD, as well as with serum Hcy levels, in the Brazilian population.

## MATERIAL AND METHODS

### Subjects

In this case-control study, a convenience sample of time and place was consecutively enrolled comprising 270 unrelated participants of both sexes aged from 37 to 76 years. Of these, 113 were healthy individuals seen at the Londrina Regional Blood Center, and 157 were patients diagnosed with PAD and receiving care at the Hemodynamics Sector of the University Hospital of Londrina, both institutions located in Londrina, Paraná, South Brazil.

Exclusion criteria were the presence of cancer or inflammatory, infectious, and autoimmune diseases, and use of folic acid and vitamin B12. The study protocol was approved by the Ethics Committee at our institution: Universidade Estadual de Londrina Human Research Ethics Committee CAAE: 78870317.8.0000.5231, Opinion number 2.400.074, of November 27, 2017. All of the individuals invited to participate were informed in detail about the research and gave written Informed Consent. All procedures were conducted in accordance with the principles outlined in the Declaration of Helsinki.

### Demographic, clinical, and laboratory data

An ABI < 0.9 determined by continuous wave Doppler was the criterion used to diagnose PAD. Continuous wave Doppler examination was performed with a DF-7001 VN Portable Vascular Doppler machine (Medpej™, Ribeirão Preto, São Paulo, Brazil). The ABI measurement technique was performed according to the American College of Cardiology/American Heart Association (ACC/AHA) guidelines for the management of patients with PAD.^21^ The ABI values were calculated by dividing the higher of the two ankle systolic pressures in one leg by the higher brachial artery systolic pressure.^22^ Subsequently, patients with PAD were categorized into five groups according to their severity using the Fontaine clinical classification, as follows: I- Asymptomatic, IIa- Mild claudication, IIb- Severe claudication, III- Pain at rest, and IV- Trophic injury.^23^ The PAD group was also evaluated for severity and anatomical distribution using anatomoradiological categories according to the Inter-Society Consensus for the Management of PAD (TASC II), with separate classifications for the aortoiliac and infra-inguinal segment (in this study, the infra-inguinal segment is referred to as the femoropopliteal and includes the popliteal-tibial segment).^23^ Each patient’s TASC category was defined by a vascular surgeon after analyzing an arteriography (Siemens™, Berlin and Munich, Germany) that was performed in the University Hospital of Londrina’s hemodynamics suite.

Anthropometric measurements (weight, height, and waist circumference) were obtained and the body mass index (BMI) was calculated. Systemic blood pressure was also measured, followed by filling out a questionnaire with data on age, ethnicity (self-reported as Caucasian or non-Caucasian), comorbidities, smoking, and use of medications. The data related to the clinical and anatomical classification of the disease and the patient’s surgical history were provided by a vascular surgeon or accessed in the medical records. Diagnoses of systemic arterial hypertension (SAH), dyslipidemia, and type 2 diabetes mellitus (T2DM) were made according to guidelines from the European Society of Hypertension/European Society of Cardiology,^24^ the Third Report of the National Cholesterol Education program,^25^ and the American Diabetes Association,^26^ respectively. Data on presence of other comorbidities, alcoholism, sedentary life style, and smoking were also recorded.

Venous blood (10 mL) was drawn after 8 hours of fasting with no anticoagulant and with EDTA anticoagulant using Vacutainer System™ tubes (Becton-Dickinson™, New Jersey, U.S). The plasma, serum, and buffy coat were stored at -80°C until analyses were conducted. Serum levels of Hcy, vitamin B12, and folate were determined by chemiluminescence microparticles assay (Architect i2000SR™, Abbott Laboratory™, Abbott Park, IL, USA).

### MTHFR C677T genotyping

Genomic DNA was extracted with a Biopur™ DNA extraction kit (Biometrix Diagnóstica™, Curitiba, Paraná, Brazil) using a 200 µL volume of buffy coat and 70° C elution buffer temperature. DNA samples were quantified using a NanoDrop 2000c™ spectrophotometer (Thermo Scientific™, Waltman, MA, USA) at 260 nm and purity was assessed by measuring the 260/280 nm ratio. The *MTHFR* 677C>T (rs1801133) variant was identified using real time polymerase chain reaction (qPCR) through the TaqMan™ (Thermo Fisher Scientific,™ Waltham, Massachusetts, EUA) method. A validated assay (C_32060205_10, Life Technologies Corporation, Carlsbad, CA, USA) with specific primers and fluorescent probes was used for genotype determination [VIC/FAM] GGC AAA CAA TAA ATG TAATAG TAG G[C/T]A AAT TTG TGC TAT GTT AGA GGT CTT). The fluorescence levels of the qPCR products were evaluated using Quantum Studio VI™ (Applied Biosystems,™ Foster City, CA, USA).

### Statistical analysis

The sample size for the study was calculated with G*Power software using the logistic case control sample size package. Assuming power = 0.90 and 95% of confidence, the sample size necessary for the study was 270 individuals (157 patients and 113 controls). Analysis of contingency tables (χ^2^ or Fisher’s exact test) was employed to check the associations between categorical variables and diagnostic groups. Categorical variables were expressed as absolute number (n) and percentage (%). We assessed the differences between groups for continuous variables using the Mann-Whitney test and expressed them as median and interquartile range 25.0%-75.0% (IQR). Genotype and allele frequencies were compared with chi-square tests. Odds ratios (OR) were calculated with a 95% confidence interval (95% CI). Binary logistic regression analysis was performed to assess the effect of the genetic variants in the study groups and OR and 95% CI were calculated. A significance level of p<0.05 was adopted for all statistical tests. The number of samples was sufficient for 80.0% power. Statistical analyses were performed using IBM SPSS for Windows version 24 (SPSS, Inc., Chicago, IL, USA).

## RESULTS

The study enrolled 60 (38.2%) females and 97 (61.8%) males with PAD and a median age of 69 years (IQR: 62-67). PAD patients were older (p <0.001) and had higher levels of Hcy than controls after adjustment for age, sex, ethnicity, and BMI (p=0.020) (Table 1). When Hcy values were adjusted for other major variables associated with high levels of Hcy, such as folate, vitamin B12, kidney disease, alcoholism, sedentary life style, and smoking, the differences between the groups’ Hcy values did not remain significant (p>0.05). Other characteristics of the PAD patients were recorded, such as 37 (24.2%) were smokers, 68 (44.4%) were ex-smokers, 98 (71.5%) were diagnosed with T2DM, 73 (47.4%) were diagnosed with dyslipidemia, 114 (74%) were diagnosed with SAH, and 58 (39.2%) had HHcy (Hcy ≥15 µmol/L). Moreover, 31 (19.9%) patients underwent endovascular revascularization, 66 (42.3%) had conventional surgery, and 59 (37.8%) adopted conservative therapy (data not shown).

**Table 1:** Sociodemographic and clinical data of peripheral arterial disease (PAD) patients and controls from the Brazilian population.

	**Control (n=113)**	**PAD (n=157)**	p value*
Age (years)	43 (37 – 48)	69 (62 – 76)	<0.001
Sex (Female/Male)	54 (47.8)/59 (52.2)	60 (38.2)/97 (61.8)	0.116
Ethnicity (C/NC)	88 (77.9)/25 (22.1)	109 (69.4)/48 (30.6)	0.123
BMI (kg/m^2^)	25.81 (22.95 – 28.80)	25.31 (21.64 – 29.73)	0.355
Hcy (µmol/L)**	9.91 (8.47 – 11.30)	13.66 (10.61 – 16.74)	0.020
Folate (ng/mL)	5.4 (3.9 – 7.5)	7.3 (5.1 – 10.6)	0.242
Vitamin B12 (pg/mL)	320 (230-450)	316 (236 – 440)	0.885

All results of Mann Whitney test. *x*^2^: results of analyses of contingency tables. Continuous variables were expressed as median and interquartile range (25%-75%) and categorical variables were expressed as absolute number (n) and percentage (%). PAD: peripheral arterial disease; C: Caucasian; NC: Non-Caucasian; BMI: Body mass index. Hcy: Homocysteine.

* Adjusted for age, sex, ethnicity, and body mass index.

** When the Hcy values were adjusted for folate, vitamin B12, kidney disease, alcoholism, sedentary life style, and smoking, the differences between Hcy values did not remain significant (p>0.05).

**Figure 1:**
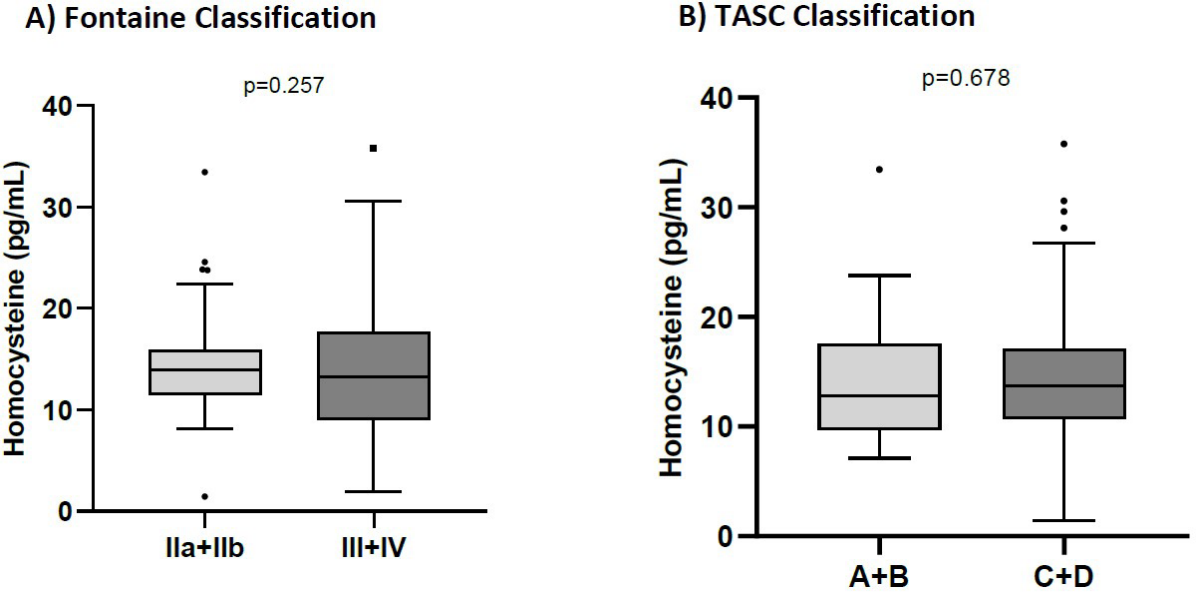
Serum homocysteine levels in patients with peripheral arterial disease (PAD) according to Fontaine and Inter-Society Consensus for the Management of Peripheral Arterial Disease (TASC) classifications (A) Fontaine classification; (B) TASC II classification. Data evaluated with the Mann-Whitney test and expressed as median and interquartile range (25%-75%). All data were adjusted for age, ethnicity, BMI, folate, vitamin B12, kidney disease, alcoholism, sedentary lifestyle, and smoking. I: asymptomatic; IIa: mild claudication; IIb: severe claudication; III: pain at rest; IV: trophic injury.

Table 2 shows the clinical characteristics of the PAD patients according to Fontaine classification and anatomical location. No patients in the study had Fontaine class I disease. There were 35 (22.4%), 33 (21.2%), 11 (7.0%), and 77 (49.4%) patients with Fontaine classes IIa, IIb, III, and IV, respectively. Fontaine classes IIa and IIb were analyzed together, since the prognosis and treatment of choice are similar, making a single group containing 68 (43.6%) patients. Similarly, classes III and IV were also analyzed together, resulting in a group containing 88 (56.4%) individuals. Forty (25.8%) patients had disease in the aortoiliac segment and 115 (74.2%) patients were free from disease in that region. In relation to the infra-inguinal segment, referred to as the femoropopliteal in this study, 134 (86.5%) patients had disease in this territory. In common with the patients with aortoiliac disease, the majority of femoropopliteal patients had more advanced disease, with 36 (23.2%) graded TASC C and 71 (45.8%) graded TASC D. We also diagnosed 19 (12.3%) patients with concomitant disease in the aortoiliac and femoropopliteal territories. When we grouped together cases for which the treatment of choice is similar, without distinction by arterial anatomy, we observed 23 (14.8%) patients graded as TASC A + B and 132 (85.2%) graded as C + D. However, Hcy levels did not differ according to clinical stage of PAD (p=0.257) (Figure 1A) or anatomoradiological categories (p=0.678) (Figure 1B). All data were adjusted for age, ethnicity, BMI, folate, vitamin B12, kidney disease, alcoholism, sedentary life style, and smoking.

**Table 2:** Clinical characteristics of peripheral arterial disease (PAD), according to Fontaine classification and anatomical location.

**Characteristics**	**Patients with PAD (n=157)**
Fontaine classification*	
I - asymptomatic	0 (0.0)
IIa - mild claudication	35 (22.4)
IIb - severe claudication	33 (21.2)
III - pain at rest	11 (7.0)
IV - trophic injury	77 (49.4)
Intermittent claudication (IIa + IIb)	68 (43.6)
Critical limb ischemia (III + IV)	88 (56.4)
Aorto-iliac (No/Yes)**	115 (74.2)/40 (25.8)
TASC A	7 (4.5)
TASC B	5 (3.2)
TASC C	12 (7.7)
TASC D	16 (10.3)
Femoropopliteal (No/Yes)*	21 (13.5)/134 (86.5)
TASC A	9 (5.8)
TASC B	17 (11.0)
TASC C	36 (23.2)
TASC D	71 (45.8)
Aorto-iliac + femoropopliteal (No/Yes)**	136 (87.7)/19 (12.3)
All patients without distinction by arteries involved: TASC A+B/C+D**	23 (14.8)/132 (85.2)

Categorical variables were expressed as absolute number (n) and percentage (%). PAD: Peripheral arterial disease; CC: homozygote genotype for the C allele; TT: homozygote genotype for the T allele; CT: heterozygote genotype for the T and C allele; TASC: Inter-Society Consensus for the Management of Peripheral Arterial Disease.

* This variable was not recorded in one patient.

** This variable was not recorded in two patients.

Table 3 shows the frequencies of the *MTHFR* 677C>T variant alleles and genotypes in the PAD patients and controls. The groups did not differ in terms of allele or genotype distributions (p>0.05), after adjustment for sex, ethnicity, age, and BMI. Table 4 shows the data related to the dominant and recessive MTHFR 677C>T statistical analysis models for the PAD subset. The subsets differed by ethnicity in both models. In addition, Hcy serum levels were higher in patients with the CT+TT genotypes than in those with the CC genotype (p=0.008, dominant model), while patients with the TT genotype had higher Hcy levels when compared with those with the CC+CT genotypes (p=0.019, recessive model), independently of sex, ethnicity, age, BMI, folate, vitamin B12, dyslipidemia, T2DM, hypertension, and smoking. The higher Hcy levels observed in patients with PAD and the T allele compared to those without this minor allele remained significant after adjustment for all these possible confounding variables.

**Table 3:** Distribution of frequencies of genotypes and alleles of *MTHFR* 677C>T variant in different genetic models in patients with peripheral arterial disease (PAD) and controls from the Brazilian population.

**Genetic model**		**Controls (n=113)**	**PAD (n=157)**	**OR (95% CI)**	p-value*
Allelic	C	156 (69.0)	209 (66.6)	Reference	-
	T	70 (31.0)	105 (33.4)	1.120 (0.778 – 1.628)	0.576
Codominant	CC	54 (47.8)	76 (48.4)	Reference	-
	CT	48 (42.5)	57 (36.3)	1.230 (0.423 – 3.579)	0.704
	TT	11 (9.7)	24 (15.3)	3.415 (0.655 – 17.79)	0.145
Dominant	CC	54 (47.8)	76 (48.4)	Reference	-
	CT+TT	59 (52.2)	81 (51.6)	1.484 (0.536 – 4.105)	0.447
Recessive	CC+CT	102 (90.3)	133 (84.7)	Reference	-
	TT	11 (9.7)	24 (15.3)	3.029 (0.660 – 13.91)	0.154
Overdominant	CC+TT	65 (57.5)	100 (63.7)	Reference	-
	CT	48 (42.5)	57 (36.3)	0.921 (0.346– 2.454)	0.870

χ^2^: results of analyses of contingency tables. Data were expressed as absolute number (n) and percentage (%). Bold values represent statistically significant values. PAD: peripheral arterial disease; CC: homozygote genotype for the C allele; TT: homozygote genotype for the T allele; CT: heterozygote genotype for the C and T allele.

* Adjusted for age, sex, ethnicity, and body mass index (BMI). OR: odds ratio; CI: confidence interval; C: major allele; T: minor allele.

**Table 4:** Results of analysis of dominant and recessive models of associations between the *MTHFR* 677C>T variant and exploratory variables in patients with peripheral arterial disease (PAD).

**Variable**	**CC (n=76)**	**CT+TT (n=81)**	p value*	**CC+CT (n=133)**	**TT (n=24)**	p value*
Age (year)	71 (61 – 78)	68 (62 – 74)	0.321	69 (61 – 76)	71 (63 – 76)	0.963
Sex (Female/Male)	26 (34.2)/50 (65.8)	34 (42.0)/47 (58.0)	0.317	50 (37.6)/83 (62.4)	10 (41.7)/14 (58.3)	0.705
Ethnicity (C/NC)	44 (57.9)/ 32 (42.1)	65 (80.2)/16 (19.8)	0.002	88 (66.2)/45 (33.8)	21 (87.5)/3 (12.5)	0.037
BMI (kg/m^2^)	24.57 (21.26 – 27.92)	26.17 (22.43 – 30.61)	0.157	25.35 (21.67 – 29.73)	24.98 (20.31 – 29.37)	0.489
Homocysteine (µmol/L)	12.94 (10.02 – 15.98)	14.60 (10.86 – 17.83)	0.008	13.22 (10.20 – 15.97)	16.40 (13.65 – 23.77)	0.019
Folate (ng/mL)	7.1 (5.2 – 10.6)	7.5 (4.8 – 10.8)	0.945	7.5 (5.3 – 11.1)	6.3 (3.7 – 9.6)	0.126
Vitamin B12 (pg/mL)	341 (241 – 459)	299 (217 – 418)	0.202	318 (244 – 445)	276 (185 – 395)	0.596
Fontaine IIa+IIb/II+IV	32 (42.1)/44 (57.9)	36 (45.0)/44 (55.0)	0.716	56 (42.4)/76 (57.6)	12 (50.0)/12 (50.0)	0.548
TASC A+B/C+D**	10 (13.3)/65 (86.7)	13 (16.2)/67 (83.8)	0.610	20 (15.2)/112 (84.8)	3 (13.0)/20 (87.0)	0.544

Mann Whitney test. Data were expressed as median and interquartile range (25%-75%). χ^2^: results of analyses of contingency tables. Data were expressed as absolute number (n) and percentage (%). PAD: peripheral arterial disease. CC: homozygote genotype for the C allele; TT: homozygote genotype for the T allele; CT: heterozygote genotype for the C and T allele. C: Caucasian; NC: Non-Caucasian; BMI: Body Mass Index; TASC: Inter-Society Consensus for the Management of Peripheral Arterial Disease.

* Adjusted for age, sex, ethnicity, BMI, folate, vitamin B12, dyslipidemia, kidney disease, alcoholism, sedentary lifestyle, and smoking

** This variable was not recorded in two patients.

There were no associations between genotypes and Hcy serum levels in the control group (regardless of whether they were analyzed with the dominant or the recessive models). The frequency of genotypes also did not differ according to different Fontaine and TASC classifications in the PAD subset (data not shown).

## DISCUSSION

The main results of this study are that the T allele of the *MTHFR* 677C>T variant was only associated with increased Hcy serum levels in PAD patients (not in controls), independently of some variables such as age, sex, ethnicity, and BMI. This result is to be expected since all of these pathophysiological conditions, such as folic acid levels, renal failure, alcoholism, sedentary lifestyle and smoking interfere with Hcy values and are inherent to the pathophysiology of PAD. However, the aim of the present study was to assess whether there is an association between the *MTHFR* 677 C>T variant and Hcy in the subset of PAD patients and, even after adjusting for the variables possibly associated with the disease that we recorded for this group of patients and which could confound the results, Hcy values remained higher among patients with the minor T allele, regardless of the genetic model evaluated in the statistical analysis, whether the recessive model (TT versus CC+CT) or the dominant model (CT+TT versus CC). Moreover, there were no associations between genotypes and either Fontaine or TASC classification. We demonstrated that patients with PAD showed higher Hcy serum levels than controls, but did not differ according to disease severity. To our knowledge, the present study is the first to evaluate associations between the *MTHFR* 677C>T variant, Hcy levels, and the severity of PAD in Brazilian patients.

Our data are in agreement with a previous study that reported HHcy in 28-30% of patients with PAD,^13^ and underscore that increased Hcy is an important risk factor for cardiovascular diseases.^27^ Hcy may affect both atherogenesis and the resistance of the endothelium to thrombosis through different mechanisms including the direct toxic effect to endothelial cells, interference with the antithrombotic and vasodilator functions of nitric oxide,^28^ and enhancement of leukocyte adhesion and extravasation, with the potential to contribute to vascular injury.^29^ The pro-coagulant effect of modest levels of HHCy (< 100 μmo/L) was evident in patients with PAD diagnosed as intermittent claudication. In isolated platelets taken from patients with high plasma Hcy (average 19 μM), the inhibitory effect of a nitric oxide donor on platelet-fibrinogen binding was 5-fold lower than in subjects with normal Hcy (average 11 μmo/L).^28^

The *MTHFR* 677C>T (rs1801133) variant of *MTHFR* consists of a cytosine (C) to thymine (T) transition at nucleotide 677 in exon 4 and this nonsynonymous variant results in an alanine-to valine (Ala to Val) amino acid change, leading to a thermolabile enzyme with reduced activity.^29^ The reduced *MTHFR* enzyme activity and its increased thermolability lead to a decrease in 5-MTHFR and an increase in accumulation of the 5,10-MTHFR substrate and, consequently, of HHcy.^16^

Our data show that the frequencies of alleles and genotypes of the *MTHFR* 677C>T variant did not differ between the PAD patients and controls in the different genetic models that were evaluated, suggesting that this genetic factor, by itself, may not contribute to the development of PAD in our population. These results are in agreement with previous studies reported in worldwide populations^18^ as well as in the Brazilian population.^20^ However, the observed frequencies of the *MTHRF* 677C>T genotypes in the present study agree with previous studies that showed presence of the TT genotype in 5.0–15.0% of the general population and its role and impact on the Hcy levels may vary in different populations.^30-32^ In addition, we also demonstrated that patients with PAD and the TT genotype (in the recessive model) had higher Hcy levels than those with the CT + TT genotypes. Moreover, patients with PAD and the TT + CT genotypes (in the dominant model) had higher Hcy levels than those with the CC genotype, suggesting that this variant may be related to HHcy in Brazilian PAD patients. It is important to highlight that these associations were independent of sex, ethnicity, age, BMI, folate, vitamin B12, dyslipidemia, T2DM, hypertension, and smoking.

Divergent results have been reported for the association between Hcy levels and *MTHFR* 677C>T genotypes. Khandanpour et al.^19^ showed that PAD was associated with elevated levels of Hcy and the TT genotype, while another study showed that the T allele could be a protective factor against PAD^33^ and that high levels of Hcy were associated with the T allele only in those individuals with decreased levels of folate, vitamin B12, or vitamin B6.^34^ These results can be explained by the fact that folate and vitamins B12 and B6 are, respectively, substrate and cofactors in the Hcy metabolic pathway.^16^ Moreover, no significant association between the *MTHFR* 677C>T genotypes and Hcy levels was also reported.^18,35^

It is important to emphasize that our data showed that the presence of the T allele was associated with an increase in Hcy levels exclusively in the PAD patients with the CT + TT genotypes, regardless of other risk factors. PAD has complex phenotypes that depend on multiple other genetic and environmental factors, as well as epigenetic mechanisms^8^ that could modulate Hcy metabolism and could in part explain the high levels of Hcy among patients with PAD and the T allele of the *MTHFR* 677C>T variant. Several risk factors for PAD could regulate gene expression through epigenetic mechanisms. T2DM, SAH, Hcy levels, aging, smoking, and sedentary lifestyle exert epigenetic effects on DNA methylation and chromatin remodeling, histone acetylation, and microRNA expression and regulation.^36,37^ The reduced enzyme activity associated with the *MTHFR* 677C>T variant has been linked to decreased DNA methylation^38^ and HHcy.^39^ In turn, decreased DNA methylation may involve aberrant expression of structural and matrix proteins or reduced DNA integrity, resulting in premature aging of the vascular tissue.^40^

This study has some potential limitations. First, it has a case-control design, which does not allow inferences on causal relationships between the *MTHFR* 677C>T variant and PAD, Hcy levels, or disease severity. Second, we evaluated one *MTHFR* variant, but this gene has other single nucleotide variants, and it might be interesting to evaluate whether genetic haplotypes play a role in subjects with PAD. However, this study also has strengths, such as controlling for confounding effects in the statistical analysis.

## CONCLUSION

The results showed that the *MTHFR* 677 C>T variant was not associated with susceptibility to or severity of PAD (clinical stage and anatomoradiological categories). However, the T allele of this variant was associated with HHcy in PAD patients, but not in controls, suggesting a possible interaction between *MTHFR* 677C>T variant and presence of other genetic, epigenetic, and environmental factors associated with PAD, affecting modulation of Hcy metabolism.
